# ‘Should we laugh?’ Acoustic features of (in)voluntary laughters in spontaneous conversations

**DOI:** 10.1007/s10339-023-01168-8

**Published:** 2023-11-23

**Authors:** Valéria Krepsz, Viktória Horváth, Anna Huszár, Tilda Neuberger, Dorottya Gyarmathy

**Affiliations:** 1 HUN-REN Hungarian Research Centre for Linguistics, Benczúr U. 33, 1068 Budapest, Hungary; 2 Humboldt-Universität zu Berlin, Unter den Linden 6, 10117 Berlin, Germany

**Keywords:** Laughter, Spontaneous speech, Voluntary–involuntary, Speech production, Speech perception, Non-verbal feature

## Abstract

Laughter is one of the most common non-verbal features; however, contrary to the previous assumptions, it may also act as signals of bonding, affection, emotional regulation agreement or empathy (Scott et al. Trends Cogn Sci 18:618–620, 2014). Although previous research agrees that laughter does not form a uniform group in many respects, different types of laughter have been defined differently by individual research. Due to the various definitions of laughter, as well as their different methodologies, the results of the previous examinations were often contradictory. The analysed laughs were often recorded in controlled, artificial situations; however, less is known about laughs from social conversations. Thus, the aim of the present study is to examine the acoustic realisation, as well as the automatic classification of laughter that appear in human interactions according to whether listeners consider them to be voluntary or involuntary. The study consists of three parts using a multi-method approach. Firstly, in the perception task, participants had to decide whether the given laughter seemed to be rather involuntary or voluntary. In the second part of the experiment, those sound samples of laughter were analysed that were considered to be voluntary or involuntary by at least 66.6% of listeners. In the third part, all the sound samples were grouped into the two categories by an automatic classifier. The results showed that listeners were able to distinguish laughter extracted from spontaneous conversation into two different types, as well as the distinction was possible on the basis of the automatic classification. In addition, there were significant differences in acoustic parameters between the two groups of laughter. The results of the research showed that, although the distinction between voluntary and involuntary laughter categories appears based on the analysis of everyday, spontaneous conversations in terms of the perception and acoustic features, there is often an overlap in the acoustic features of voluntary and involuntary laughter. The results will enrich our previous knowledge of laughter and help to describe and explore the diversity of non-verbal vocalisations.

## Introduction

Non-verbal features have a ubiquitous role in human vocal communication. The non-verbal features of social interaction have been partly ignored in the past decades due to the lack of adequate understanding of their social and cognitive effects on communication, as well as some limitations regarding the technical possibilities of the studies. Nowadays, however, the study of non-verbal features of communication has become one of the prior issues of robotics, artificial intelligence and the diagnosis of various diseases (Due 2019; Hietalahti 2021). Besides gestures, eye-gaze, head, hand and (upper) body movement, laughter carries essential information and plays an important role in the turn-taking system, as well. Apart from the visual features, non-verbal vocalisations like cough, humming, audible inhalation express information regarding the emotional state of the current speaker, as well as the intentions in the local communication event (Danner et al. 2021).

Laughter is one of the most common non-verbal features in communication. However, the frequency values may change based on the fact that laughter is not a homogenous group, the occurrence of laughter shows great variations depending on the topic, the level of acquaintanceship, the hierarchy between the participants, the personality of the speakers and in addition, it shows culture-dependent features (cf. Vettin and Todt 2005; Holmes and Marra 2002; Mazzocconi et al. 2016; Neuberger 2012).

The results corroborated that most laughter can be found in social interactions like conversation; however, they rarely occur during jokes (20% of all cases according to an experiment, Provine 1996), but more likely as signals of the agreement, the empathy, the belonging to the group, etc. Laughter may occur in pauses or at the end of the phrases more often in general, suggesting that placement of the phenomenon is coordinated by neurological processes that match laughter and speech (Wild et al. 2003).

It was questionable whether the occurrence of laughter depends on the speech situation; thus, the frequency was compared in spontaneous dialogues (48 h of conversations of 10 participants, six women and four men in familiar surroundings on several days) and in an experimental situation (at the university in the framework of an undergraduate course) in the case of Vettin and Todt’s (2004) research. Results showed that there was no difference in the frequency of the laughter in dialogues when the participants were acquaintances and strangers. The authors assumed that high frequency of laughter in dialogues with strangers is a result of communicative rules which are for avoiding misunderstanding and misinterpretation of utterances. In addition, laughter in dialogues with strangers contribute to establishing new relations that is corroborated by the results of other researches, as well (c.f. Grammer 1990; Devereux and Ginsburg 2001).

Not only the frequency of laughter, but also their other acoustic features reveal great variety throughout the different experiments. Although it has been a constant view that the components of laughs are conceived predominantly as vowel-like outbursts (such as Darwin 1872/1998; Ruch 1993; Nwokah et al. 1999), there may be some variation between the individual sounds that make up laughter (Urbain and Dutoit 2011; Ruch and Ekman 2001). It has been now shown that the structure of laughter is much more complex than it was hypothesised earlier. Laughter was acoustically characterised by Provine (1996) as follows: short vowel-like notes (75 ms long in general), which were recurred several times regularly. These notes separated from each other by 210-ms-long unvoiced aspiration. The mean fundamental frequency was about 502 Hz in the female’s laughter, while 276 Hz in the case of male laughter. The intensity of laughter decreases from the beginning to the end. According to another study on laughter realisations (Vettin and Todt 2004), the mean f0 of laughter bouts was found to be 171 Hz in the male group (106–355 Hz), while 315 Hz in the female group (117–735 Hz). The duration of two-subjects’ laughter was analysed by Bickley and Hunnicutt (1992). Due to the fact that a given type of laughter sounds like a sequence of breathy CV syllables, the average duration of a laugh syllable was investigated. Results showed that this value was 204 ms for one speaker and 224 ms for the other speaker. The mean number of syllables was 6.7 in one speaker’s laugh, while 1.2 syllables in the other’s laugh. In a later study, they found a mean duration of laughter of 798 ms in the female’s group, while it was on average 601 ms in the male’s group (Rothgänger et al. 1998). The mean value of the fundamental frequency in the female’s group varied between 160 and 502 Hz, while between 126 and 424 Hz in the male’s group (Bachorowski et al. 2001).

### Laughter types

However, the previous research managed laughter as one group; Scott et al. (2014) distinguished between two types of laughter. Different research labelled them in different ways: voluntary/involuntary (Scott et al. 2014; Chen 2018) or voluntary/evoked (Scott et al. 2014), spontaneous, authentic/fake (Lavan et al. 2016), social/spontaneous (Shochi et al. 2017), spontaneous/volitional (Bryant et al. 2018; Kamiloğlu et al. 2021), mirthful/polite (Tanaka and Campbell 2014; Sabonyté 2018), authentic/acted (Anikin and Lima 2018)—depending on the framework of the given analyses, in some research used as synonyms. However, the two concepts differed not only in their names, but often in their definitions as well.

Some studies have selected the sound samples for a perception test on the basis of a preliminary grouping:

Similarly, Shochi et al. (2017) investigated the types of laughter in regard to their voluntariness. Firstly, 3 Japanese males and 4 French subjects were asked to listen to 254 laughter that were collected from 12 spontaneous conversations during online video games and decide whether it was social (‘the person is laughing to maintain the communication with the other (e.g. embarrassed laughter, polite laughter, cynical laughter…’) or spontaneous (‘the person is laughing in a spontaneous manner to an external event (e.g. a funny clip)’)). Additionally, ‘I don’t know’ answer was also possible. Then, from altogether, 27 spontaneous and 27 volitional social laughs built up the dataset. In the perception test, 20 Japanese and 82 French native listeners listened to the stimuli and decided what kind of laughter they heard. Results showed that subjects were able to differentiate these types of laughter with about 70% accuracy based only on audio information without their context. Furthermore, acoustic analysis was also conducted regarding these two types of laughs. According to the multiple factor analysis, judgements of both French and Japanese groups were correlated with f0 features (mean and standard deviation), the total duration and the voiced segment duration. Therefore, these acoustic factors were further investigated, and results showed that the total duration of laughs is an important cue for the differentiation regarding their voluntariness. In addition, the voiced duration, the number of voiced segments and the f0 standard deviation also assist in the differentiation between spontaneous and social laughs. The variations of f0 values were higher; the total duration and the voiced segment duration were longer in the case of spontaneous laughs than social ones.

Other examinations have considered the laughs in different (genre) recordings as belonging to one group or the other:

Additionally, Bryant et al. (2018) conducted a perception test on two types of laughter as well. Participants had to decide whether the laugh was real or fake. The test contained 18 spontaneous laughs from natural conversations (real laughter) and 18 ‘fake/volitional’ laughs. The laughter regarding the first category was collected from 13 conversations, involving female friend speakers, while volitional laughter as samples for second category were recorded from women who were instructed to ‘now laugh’ with no other prompting. This task was conducted with 884 participants from six regions of the world. The overall rate of correct judgments was 64%, which was a performance significantly better than the chance level in the differentiation of real and fake laughs. Results showed that people are able to distinguish spontaneous and fake laughs, regardless of their language or culture. Laughs produced with greater intensity variability, higher pitch, and increased noisy features are considered to be spontaneous.

In another study (Kamiloğlu et al. 2021), spontaneous laughs were elicited by funny videos (they laughed in response to self-selected humorous recordings), while participants were instructed to politely laugh at unfunny jokes for collecting volitional laughter from the similar people. In total, almost eight hundred laughter samples were collected from Dutch and Japanese speakers. Then, 20 Dutch and 18 Japanese participants were asked to answer two questions: ‘Do you think this was a genuine or a polite laugh?’ and ‘Did this laugh sound authentic or not?’—they were asked to choose the yes or no response. Sixteen clips (eight Dutch, eight Japanese; laughter type and gender balanced for each group) that were most accurately discriminated as spontaneous versus volitional and that were judged as most authentic were selected as stimuli for the main experiment. Statistical analysis corroborated previous results: spontaneous laughs had higher rates of intervoicing interval, longer duration, increased f0, F1 and F2 means, lower amplitude variability, higher values of spectral centre of gravity and reduced harmonics-to-noise ratios. These 16 laughs were the stimuli of the main experiment. Participants were asked to hear decontextualised laughs and then decide (i) whether it was spontaneous or volitional; (ii) whether the laughing person was from their own or foreign cultural group. Participants also rated the positivity of each stimulus on a 7-point Likert scale. Results showed that both Dutch and Japanese participants rated spontaneous laughter as more positive than volitional ones. However, no difference was found in the accuracy of group membership identification from spontaneous versus volitional laughter.

Other analyses have selected the two different types of laughter from the same social context:

Lavan et al. (2016) conducted an experimental study on the acoustic features and perceptual judgement of volitional and spontaneous laughter. Female speakers were asked to produce both spontaneous (‘genuine amusement laughter’) and volitional (‘voluntary, controlled’) laughter. Then, 72 stimuli were selected for a perception test. Nineteen participants were asked to rate the valence and arousal of the stimuli on a 1–7 Likert scale. According to the acoustic analysis, significant differences were found between volitional and spontaneous laughter for most of the measured acoustic parameters: longer total duration, shorter burst duration, higher f0 mean, higher f0 minimum and maximum, a larger f0 variability, a higher percentage of unvoiced segments and lower mean intensity were measured in the case of spontaneous laughter compared to volitional ones. However, they did not find differences between the two laughter types in f0 range, harmonics-to-noise ratio (HNR) and spectral centre of gravity. Furthermore, the results of the perceptual experiment showed that spontaneous and volitional laughter were perceived as being different in arousal, valence, and authenticity; therefore, participants were able to distinguish between these two types of laughter. Combinations of the laughter’s total duration, spectral centre of gravity, and f0 mean were the most prevalent predictors for ratings of spontaneous laughs. In contrast, in the case of volitional laughs, HNR was found to be the most frequent predictor for affective ratings. Results showed that volitional laughs, with a lower HNR, appeared to be more authentic and more positive.

In another study, 100 samples of non-overlapping spontaneous, mirthful and polite laughter were collected from daily conversations and TV talk shows (Sabonytė 2018). Thirty stimuli were used; 30 respondents were asked to label the type of laughter after hearing the recordings with and without the context. Acoustic features of the two types of laughter were analysed. Data showed significant difference in duration between mirthful and polite laughs, but neither in intensity, in f0, F1, F2, in shimmer and jitter values. Bouts of mirthful laughter were longer than bouts of polite laughter of the same number of structures: polite laughter contained one, whereas mirthful laughter consisted of one and more bouts. In the case of polite laughter, the most common form was a two-syllable laughter bout, the mirthful laughter may consisted of more than one bout; the bouts of this type of laughter consisted of one to fifteen syllables (the most common samples are of three or four syllables). In addition, differences were found between the accuracy of cluster analysis and human perception in distinction of these laughter types.

Differentiation between acted and authentic emotional non-verbal vocalisation was analysed (Anikin and Lima 2018). The judgement task was conducted online. The participants were asked to listen to various sounds (from seven different corpora) and decide whether they were real (authentic) or fake (pretending). Results showed that the accuracy of authenticity detection varied regarding the emotional category. Authentic non-verbal vocalisations of fear, anger and pleasure were much more likely to be deemed as authentic as posed vocalisations. The authentic laughs were perceived as authentic in 67% of all cases, and they occurred with higher pitch, larger pitch variability and lower harmonicity than fake ones.

Other studies also investigated the influence of the relation of the speakers on laughter perception (Farley et al. 2022). Laughter stimuli were obtained from telephone calls of 27 callers talking to their romantic partner and a close same-sex friend. Fifty-two samples were selected for the study: in the first task, these laughter had to be judged by the 50 raters regarding pleasantness. In the second task, listeners had to decide whether the laughter was directed towards a friend or a romantic partner. Results showed that listeners were able to identify them in a higher proportion than the chance level (57%). Furthermore, laughter directed at romantic partners were judged to be less pleasant-sounding than those directed at friends. Additionally, in the second part of the study, the eight, most prototypical laughs were selected. Participants had to judge laughter samples regarding spontaneity using bipolar scales (e.g. ‘loud/soft’, ‘natural/forced’, ‘breathy/not breathy’) and regarding vulnerability. Laughter directed at friends were judged to be louder, more masculine, natural-sounding, ‘changing’, mature-sounding, more dominant, and less breathy than those directed at romantic partners. The gender of the speakers also affected judgements significantly: laughter samples from male speakers received higher ratings for masculinity and coldness, while female laughter samples were higher rated for loudness, naturalness, changing, maturity, relaxed, and dominance.

Beside the research focusing on the judgements of laughter types, another group of research aimed at the **automatic classification** of different kinds of laughs. The laughter detector developed by Campbell et al. (2005) can automatically recognise four laughter types based on the speaker’s affective state and their segmental composition (voiced laugh, chuckle, ingressive breathy laugh, nasal grunt). The identification rate was greater than 75%. In Galvan et al. (2011), automatic recognition based on vocal features also achieved high accuracy scores (70% correct recognition) when discriminating five types of acted laughter: happiness, giddiness, excitement, embarrassment and hurtful.

By social function, samples of laughter were divided into five groups: mirthful, politeness, embarrassment, derision and others (Tanaka and Campbell 2011). In natural communication, the most frequent types of social laughter seemed to be polite and mirthful (Tanaka and Campbell 2011, 2014; Sabonytė 2018). In order to distinguish between different types of laughter, and based on phonetic characteristics (voiced, ingressive, chuckle, nasal), Tanaka and Campbell (2011) used HMM with the following spectral features: MFCC, RMS power, and delta, power, and achieved a prediction accuracy of 86.79%.

In another study, Tanaka and Campbell (2014) categorised laughs into either polite or genuinely mirthful categories (based on the majority vote of 20 observers). They determine the main contributing factors in each case by statistical analysis of the acoustic features, principal component analysis and classification tree analysis. SVM was used to predict the most likely category for each laugh in both speaker-specific and speaker-independent manner. Better than 70% accuracy was obtained in automatic classification tests.

Through the investigation of laughter-related body movements, five laughter states (hilarious, social, awkward, fake, and non-laughter) were distinguished automatically by Griffin et al. (2015).

The automatic detection and classification of laughter occurrences can be beneficial in a number of ways. It could be used in automatic speech recognition (ASR) systems, reducing the word error rate by identifying non-speech sounds. It can be helpful in searching for videos with humorous content. Detection of users’ emotional state from various modalities (body movements, facial expressions, speech) and production of emotional displays can be used in design of human–computer interaction (HCI). Automatic detection of laughter can be useful for detecting the user's affective state and conversational signals such as agreement. Thus, it may facilitate affect-sensitive multimodal human–computer interfaces. Virtual/embodied agents could be made more natural (human-like) using natural-sounding synthesised laughter.

Previous research has therefore made several findings regarding the categorisation of laughter. The previous research examined the different realisations of laughter from many aspects: in the introduction part, we focused on the results obtained based on three main aspects: the production, the perception and automatic categorisation and classification. However, drawing general conclusions is made more difficult by the fact that the individual studies differed in many respects: Some research contrasted spontaneous laughter with fake (Lavan et al. 2016), social (Shochi et al. 2017), volitional (Kamiloğlu et al. 2021), while other examinations distinguished between voluntary/involuntary (Scott et al. 2014; Chen 2018), voluntary/evoked (Scott et al. 2014), or mirthful/polite (Tanaka and Campbell 2014; Sabonyté 2018). On the one hand, these researches defined the groups of laughter differently. On the other hand—as well in close connection with this fact—they also use different methodologies with regard to the data collection: real laughs were collected from ‘natural, spontaneous’ recordings (see Shochi et al. 2017; Bryant et al. 2018; Sabonytė 2018) or during funny videos (Kamiloğlu et al. 2021), ‘fake’ laughter were forced in an artificial way, for example, they asked the speakers to show how would they laugh at an unfunny joke in a polite way (Kamiloğlu et al. 2021), professional actors were requested to produce different types of laughter (Szameitat et al. 2009), or participants were called just to laugh (Bryant et al. 2018), while in other studies both of the laughter were recorded during video games. In addition, differences have been found in respect with, for example, the analysed acoustic features, measurement methods, as well. The results and the conclusions that can be drawn from them are therefore difficult to generalise and are highly limited: The results of the production and perception examination listed above—independently from the categories used to concepts, methods and/or definitions of the different kinds of laughter—found differences between their categories’ laughs acoustically and perceptually. Most of the research maintained the effect of the timing features regarding that the spontaneous laughter realised with longer duration than the samples in the other category. Although, in the case of f0 characteristics, not all research has corroborated a difference between the two groups, if they do, the mean f0 was higher in the case of the spontaneous or real laughter than as for the other category. Regarding the other parameters, such as the intensity, CoG, F1, F2, pitch, jitter, shimmer, HNR, the results of the research were often contradictory.

## The present study

It is important to highlight that research listed above mostly created artificial speech or recording situations to contrast the two laughter groups: speakers were asked to produce laughter in different ways, the laughter was recorded during video games, they were recorded from TV shows or while the speakers were watching funny videos. However, we have little information about what differences the listeners may detect between the types of laughter when both types come from a more natural form of communication, face-to-face, multi-party, offline recorded communication situations.

The question arises whether a similar distinction can be made between laughter as it was found in the previous research, even if they are recorded not in artificial circumstances, but in face-to-face three-party conversations. In contrast to previous research, the purpose of the present study is not to evaluate two predetermined groups of laughter with listeners. Our aim is rather to compare and describe those laughter groups that appear in human interactions and that were uniformly judged by listeners according to whether the participants defined them to be voluntary or involuntary. In other words, the results of the research show the acoustic characteristics of the laughter considered to be voluntary and involuntary, as well as the results of its automatic classification.

In this way, we do not get an idea of whether the speakers are able to recognise which category the given laughter belongs to (for example, according to how they were recorded), but we analyse whether a systematic difference in the acoustic characteristics can be justified in the decision of the listeners based on manual comparison and automatic classification. Additionally, our goal was to examine only laughs from natural, close to spontaneous conversations, as opposed to previous studies, because these are the most natural and most common forms of everyday verbal and non-verbal communication. The main question is, how uniformly and relying on which acoustic features do listeners consider laughter with regard to their voluntariness in conversations, without any context.

In addition, although studies of laughter have been done in many languages, to the best of our knowledge, few analyses have been done in Hungarian, and none of them have previously examined differences by voluntary–involuntary categories. The present research is therefore the first to provide data on the Hungarian language in this framework.

The present research consists of three parts using a multi-method approach: (1) a perception test on laughter related to its voluntariness, (2) an acoustic analysis comparing the acoustic structure of laughter which were considered to be voluntary vs. involuntary, (3) automatic classification of voluntary/involuntary laughter, using different algorithms.

According to our hypotheses (1), participants are able to distinguish involuntary and voluntary laughter without context, even if all of them are from both spontaneous three-party conversations. (2) There will be a difference in acoustic pattern between the two groups (involuntary and voluntary laughter). (3) With different kinds of automatic methods, it will be possible to distinguish between voluntary and involuntary laughter groups based on their acoustic characteristics.

## Methodology

### Judgement task

#### Laughter stimuli

The stimuli were collected from the Hungarian Spontaneous Speech Database (Neuberger et al. 2014). The database was developed at the Phonetic Department of the Research Institute for Linguistics, containing altogether 460 records. The majority of the recordings are annotated three levels of analysis (interpausal units, word, sound) in Praat (Boersma and Weenink 2020), including with a total of 8 subtasks: 2-party conversation, sentence reading and sentence repetition, 2 text readings, retelling 2 stories and 3-party conversation. The recordings were conducted in a silent laboratory, using an AT4040 microphone. The recordings were digitally made directly on the computer with GoldWave sound editing software, with sampling at 44.1 kHz (storage: 16 bits, 86 kbytes/s, mono). The subjects in the database are all monolingual adults from Hungary; their ages range between 20 and 90 years.

Laughs were collected from the 3-party conversational subtask produced by female speakers. The protocol of this conversational part is the following: Two fieldworkers from the institute and one subject participate in a recorded conversation. The topic was given by the first fieldworker at the beginning of the recording, regarding the subject’s job or area of interest (based on the previous two-participated parts of the recordings), e.g. New Year’s Eve, wedding experiences, job hunting, Easter and Christmas holiday, school violence, keeping pets in a flat, bringing up children in a city, cycling as a form of traffic, etc. The participants have no time to plan their speech production in advance, so these conversations are quasi-spontaneous in the sense of the speech planning, and the outcome depends on the opinion and utterances of the participants.

Forty-nine laughter from 27 female speakers were randomly selected from 25 conversations for this study. The number of the stimuli is similar to that used in the previous studies, e.g. Shochi et al. (2017) used 54, Bryant et al. (2018) used 36, while Sabonytė (2018) used 30 stimuli in their studies. Laughter segments were manually labelled by human annotators (see the transcription protocol of the Hungarian Spontaneous Speech Database; Neuberger et al. 2014). The boundaries of a laughter segment were determined based on auditive judgement supported by visual analysis of oscillogram and spectrogram in Praat. Overlapping laughter and speech laughter were excluded from this research. Isolated, individual laughs were selected, that is only those laughs were included in the material that were not preceded or followed by others' laughter. The final inhalation breath (if audibly or visually detectable) was considered part of the laugh (as in Mazzocconi and Ginzburg 2022). Laughs were automatically extracted using a Praat script written by one of the authors from the annotation of the database. The laughs were played for the subjects without any context.

### Procedure

The perception test was conducted online. Although the issue often arises that the conditions of the perceptual test are uncontrollable in digital form, Horton et al. (2011) and Germine et al. (2012) results showed that there is no significant difference when the perception test is performed online or in a laboratory. The perception test was developed in the GMS framework system especially for this study. GMS was originally a gamification content management system with numerous game-based learning projects. Now it is also used for personal tests, scientific research and assessment as well. It is completely web-based, responsive and mobile-friendly, using only HTML, CSS and JavaScript code.

**Fig. 1:**
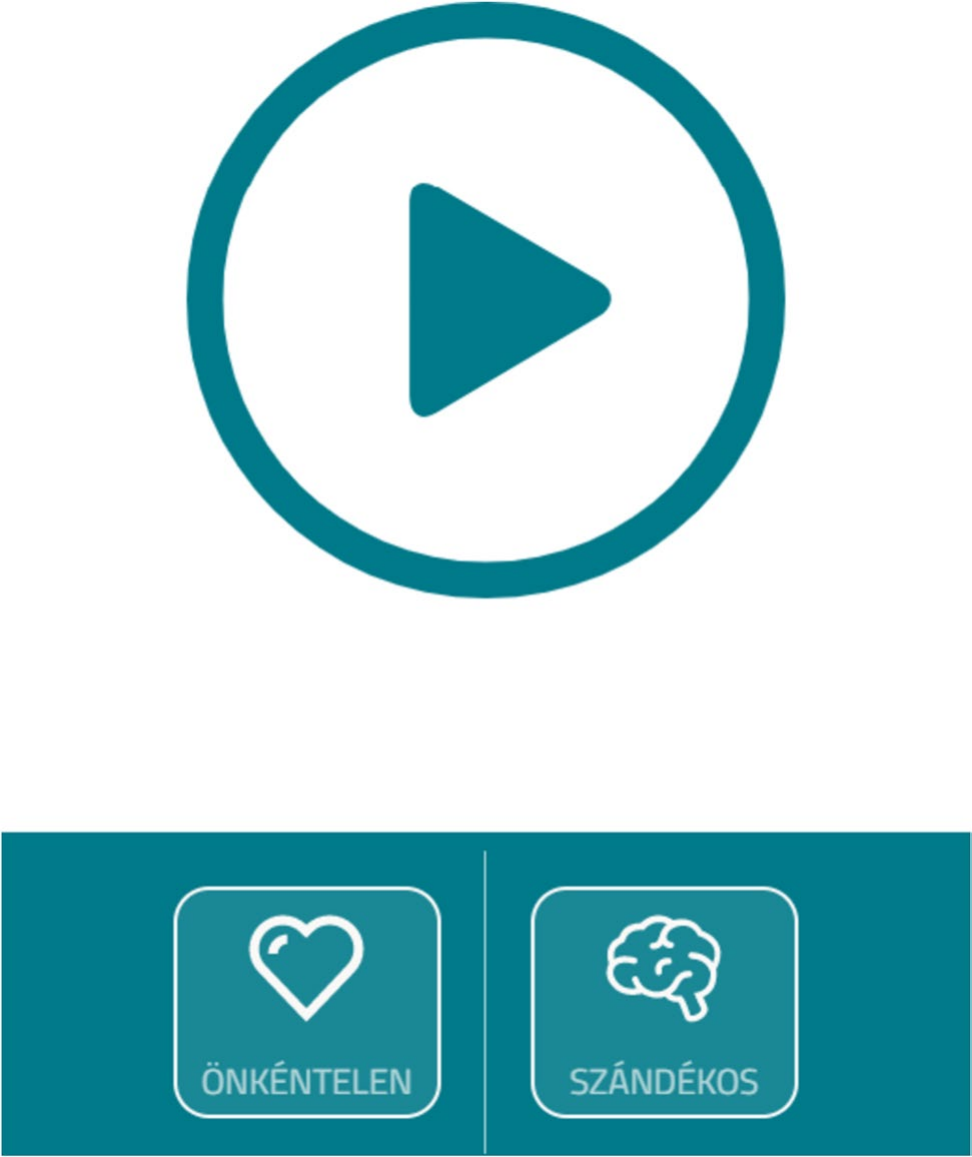
Display of the online perceptual test (involuntary laugh marked with heart motive, voluntary laugh marked with brain motive)

Forty-nine laughter stimuli were presented in a random order using an experiment software developed for this study. Participants were asked to use headphones during the test. After listening to two trial stimuli for checking the appropriate volume, they were asked to listen to each laughter and decide whether it was a voluntary or an involuntary one, clicking on the appropriate answer. In each case, they had to choose between the two options, they did not have the option to give an ‘I don’t know’ answer. The voluntary and involuntary options were labelled with text and with signs as well (with a heart and a brain motive, Fig. 1). The procedure used is similar to the judgement task of Bryant et al. (2018); however, the types of the analysed laughs were different. Participants in our task had to decide whether the particular sample seemed to be rather involuntary or voluntary, therefore, no ‘good’ or ‘bad’ answer existed, the acoustic analysis of laughs regarding the two types was based on the judgement of the participants’ majority. The reaction time of the answers was also measured by the given software to see how certain or uncertain the categorisation of the laughter was (in the case of a fast response, the confidence of the response; in the case of a slow response, the uncertainty of the response).

### Participants

One hundred and seventy people participated in the online perception test, with a mean age of 35.1 years (median 36 years, SD: 11.8 years, min.: 18 years, max.: 67 years): 136 females and 34 males. The degree of the participants varied from elementary school to master degree; however, 50% of the participants had university degrees, 29% had a high school diploma, 16% had completed post-graduate education, 2% had completed technical or vocational education and 3% had only completed primary school. The test participants were all native Hungarian speakers with no known language or hearing problems. Participation in the test was volunteer, no fee was paid. No special features were required for the participation.

### Acoustic analysis

For the acoustic analysis, sound samples of laughter were chosen which were considered to be voluntary and involuntary by at least 66.6% of listeners (cf. Shochi et al. 2017). These laughs were analysed and compared with regard to their voluntariness according to the following acoustical features:

- Total duration of laughs (ms).
- f0 mean (Hz): f0 mean was computed using the auto-correlation method in PRAAT. (Pitch floor was set at 75 Hz and the pitch ceiling at 700 Hz. The frame duration was 100 ms).
- f0 range (semitone): f0 maximum – f0 minimum converted into semitones (12*log_2_(f0_max_/f0_min_)).
- Percentage of voiced segments: percentage of the voiced parts in the total duration.
- Number of syllables (bursts): the number of vowel-like vocalic segments within laughter.
- Mean harmonics-to-noise ratio (HNR, dB): Mean ratio of quasi-periodic to non-period signal across time segments.
- Jitter (%): the average absolute difference between two consecutive periods, divided by the average period.
- Shimmer (%): the average absolute difference between the amplitudes of two consecutive periods, divided by the average amplitude.
- Intensity mean (dB): the mean of intensity values of the frames within the total laughter duration.
- Centre of gravity (CoG, Hz): the average height of frequencies in the spectrum of each laugh, the weighting of energy in the sound across the frequency range was based on the power spectrum using the built-in function of Praat.

Nonparametric Mann–Whitney tests were used for comparing these acoustic parameters of laughter and reaction time of the answering regarding voluntariness. Pearson correlation was adopted for the correlation analysis (1) between the total duration of the given laughter and the ratio of the voiced parts in the laughs and (2) between the acoustic parameters and the proportion of perceptions. Data were analysed by the R program (R Core Team 2018). We examined whether changes in each acoustic parameter were associated with a higher proportion of ratings of voluntary or involuntary for a given laughter sound pattern.

### Automatic classification

In this study, logistic regression was used to classify laughter types (*voluntary* or *involuntary)*. The latter was chosen because it is a simple algorithm for binary classification.

We applied this technique on the dataset that was extracted from the perception test. These laughter samples were categorised consistently by human listeners either as voluntary or involuntary laughter. Furthermore, we intended to improve our data source by complementing it with more samples. It is known that human-like abilities can be imparted to machines for specific tasks by means of machine learning algorithms. A classifier and semi-supervised learning algorithm were proposed that make effective use of unlabelled data to improve classification performance (Liang et al. 2007). Given the existence of labels for the laughs from the perception experiment, we applied the label propagation method to a randomly selected larger set of unlabelled laugh samples in order to label them as either voluntary or involuntary. This technique allows us to incorporate not only the knowledge of the labelled data, but also the features of the unlabelled data.

The unlabelled dataset contained 159 manually segmented laughter instances from the Hungarian Spontaneous Speech Database (Neuberger et al. 2014). The same acoustic parameters were evaluated as in the acoustic study, and these features were used for the label propagation procedure: duration, f0 mean, f0 std, HNR, local jitter, local shimmer.

Label propagation and logistic regression were carried out in Python 3.8.10 using Scikit-learn 1.0.2 (Pedregosa et al. 2011).

**Fig. 2:**
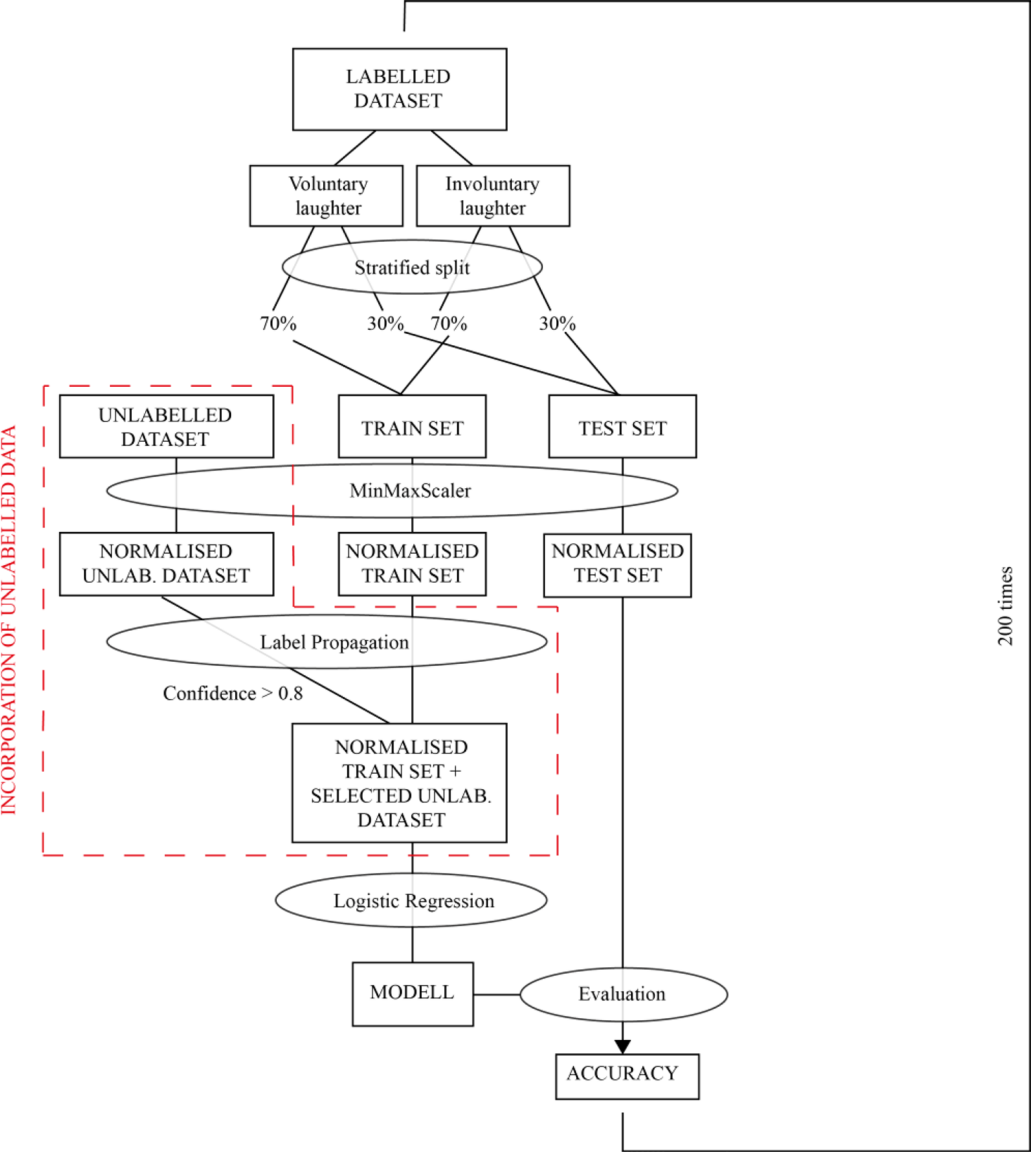
Model design

To measure the advantage of semi-supervised learning with logistic regression, a baseline with only logistic regression without additional methods was run. The labelled data was divided (labelled as either *voluntary* or *involuntary* by human listeners in the perception test) into training and test sets (Fig. 2) using stratified split method (i.e. the folds were made by preserving the percentage of samples for each class). The train–test ratio was 70–30% of the entire dataset. Features of the train set were normalised using MinMaxScaler, which scales each feature to a given range, in this case between zero and one.

Baseline logistic regression model: First, we used a logistic regression algorithm fit only on the labelled portion of the normalised training dataset and applied the trained logistic regression model to the normalised test set to establish a baseline in performance on the semi-supervised learning dataset.

Logistic regression model with label propagation: Next, the label propagation method was employed on a dataset consisting of the normalised train set and the normalised unlabelled dataset. Labels were propagated with a combination of random walk and clamping in this algorithm (Zhu and Ghahramani 2002). The goal of the label propagation technique was to predict the labels of the unlabelled samples. We selected samples in which the prediction related to the cases of confidence was higher than 0.8 and added them to a mixed training set together with the normalised train set to train logistic regression classification models on it.

Finally, the performance of the models was evaluated. The processes were run 200 times for both the baseline logistic regression model and the logistic regression model with label propagation. This ensured the variety of the test and train set composition. Most importantly, the robustness of the models was measured to see to what extent it gives different results each time it is executed (how unstable the model is). Thus, two distributions of accuracy were calculated. One-sample t-tests were conducted to test for classification accuracy above the 50% chance level, and the performance of the two models was compared to each other in pairwise *t*-test with Bonferroni’s correction (in R).

## Results

### Judgement task

**Fig. 3:**
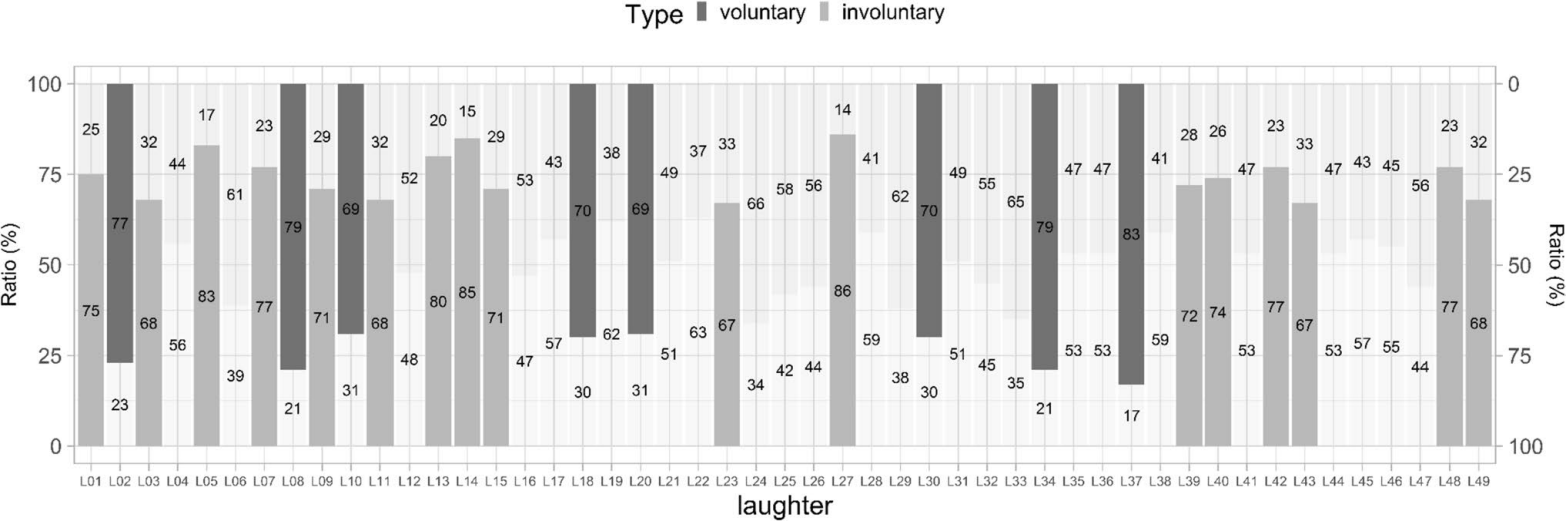
The ratio of *voluntary* and *involuntary* judgements for the 49 stimuli

Participants considered almost half of the laughter (45%) uniformly as voluntary/involuntary; however, the other half (55%) were deemed as a compound (the proportion of uniform decisions did not reach 66.6%). From those laughter that were determined as voluntary or involuntary, 46.5% were considered as to be voluntary, while 53.5% of them as to be involuntary (Fig. 3). However, the ratio of the judgements showed great idiosyncrasy across sound samples. Fifty-two percentage of all laughter samples were deemed unanimously by 50–66% of all participants, while 38% of the samples by 67–80% of the participants. Only 10% of the laughs were considered uniformly regarding voluntariness by at least 80% of the listeners.

**Fig. 4:**
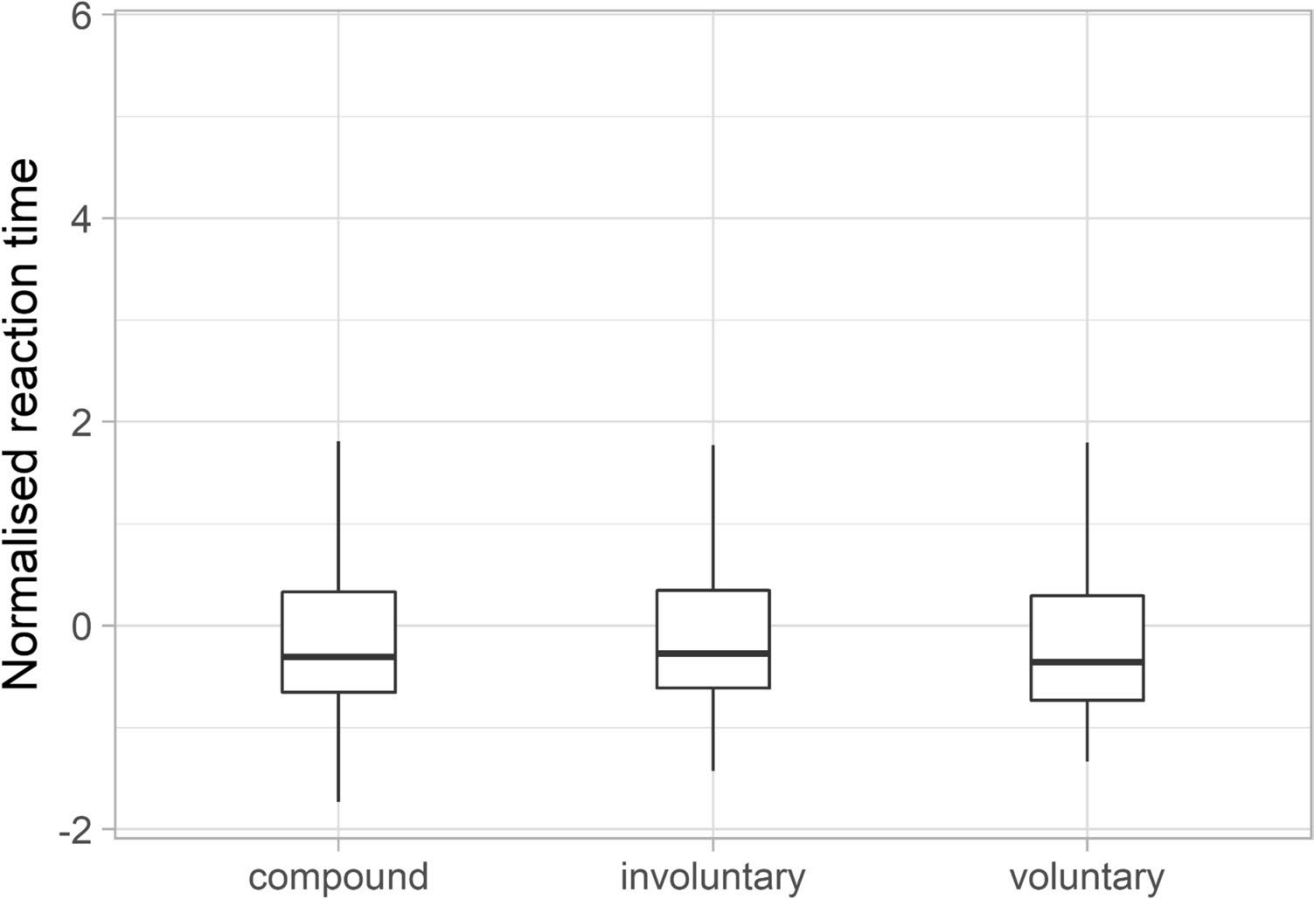
The normalised reaction time across the three categories

Reaction time was analysed regarding the results of the judgmental task. Normalised reaction time was analysed because of the participants' different tempo. (*Z*-score normalisation was applied for each participant.) Considerable difference across the three categories was not found (Fig. 4), although the mean value of the normalised reaction time in the compound category was a bit higher (+ 0.019 ± 1.049) than in the other two categories (involuntary: − 0.014 ± 0.880, voluntary:–0.049 ± 0.984). The boxplot diagram and the standard deviation values also support that there is no considerable difference in the normalised reaction time across the three categories. Normalised reaction time showed great variability across laughter samples as well: both the shortest ( − 1.730) and longest (5.680) normalised reaction times occurred in the compound category. The difference between the groups was not significant.

### Acoustic analysis

At first, the acoustic analysis was carried out to evaluate laughter, which were categorised to be voluntary/involuntary by at least 66.6% of all participants (c.f. Shochi et al. 2017).

**Fig. 5:**
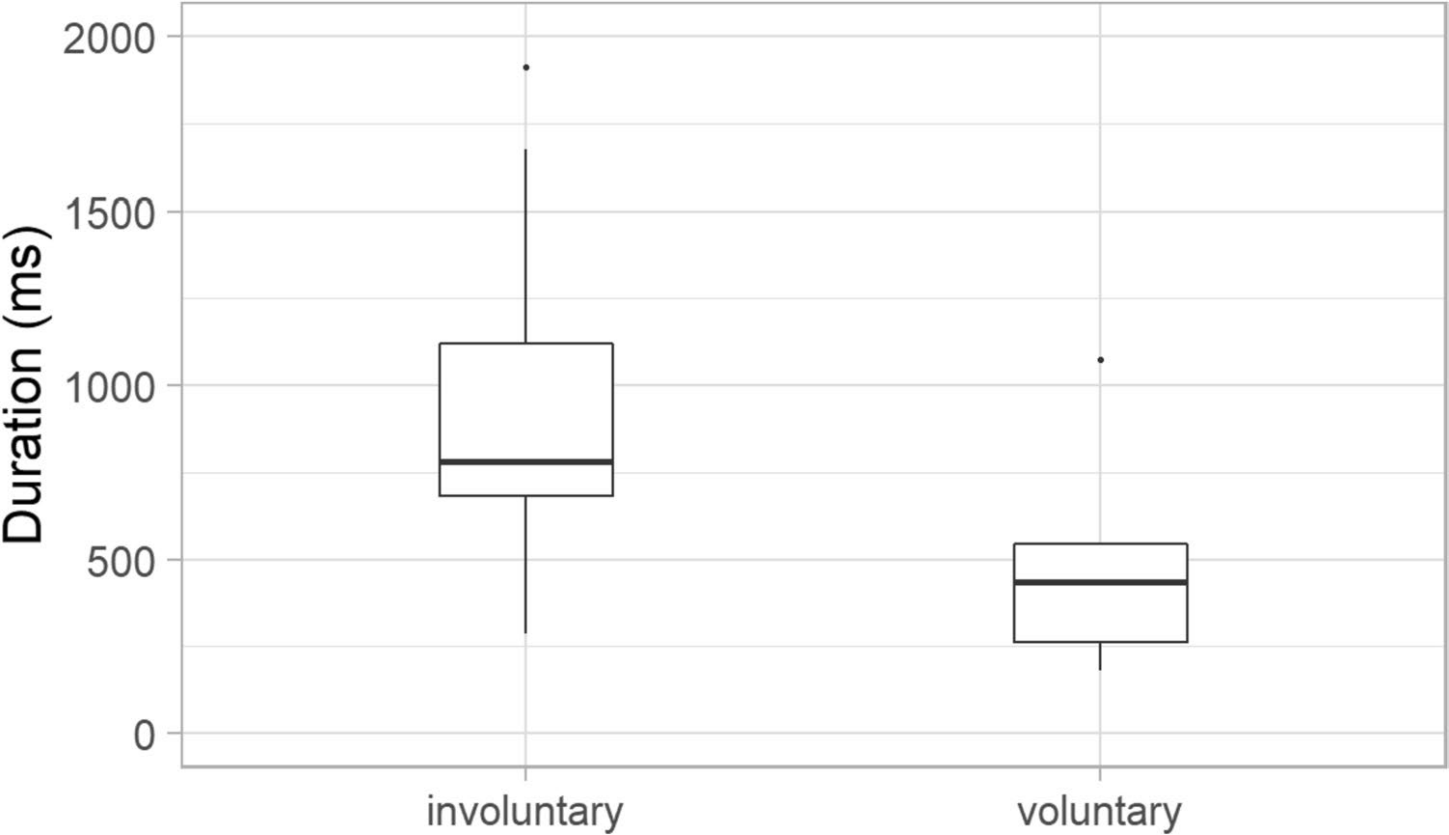
Duration of the involuntary and voluntarily laughter

The mean duration of laughs was 784 ms (SD: 434 ms). The two types of laughter can be well separated based on their duration (Fig. 5). Laughter, which was judged to be involuntary by the majority of the listeners, was longer (mean: 974 ms, SD: 429 ms) than laughter categorised to be voluntary (520 ms, SD: 407 ms). The difference was significant in the duration values between the voluntary and involuntary laughs (*Z* = − 2.117; *p* = 0.034).

The mean number of syllables (bursts) in the laughter was the same in the case of involuntary and voluntary laughs (3.4 items); however, involuntary laughter occurred with greater standard deviation (1.6 items) than voluntary ones (1.2 items).

**Fig. 6:**
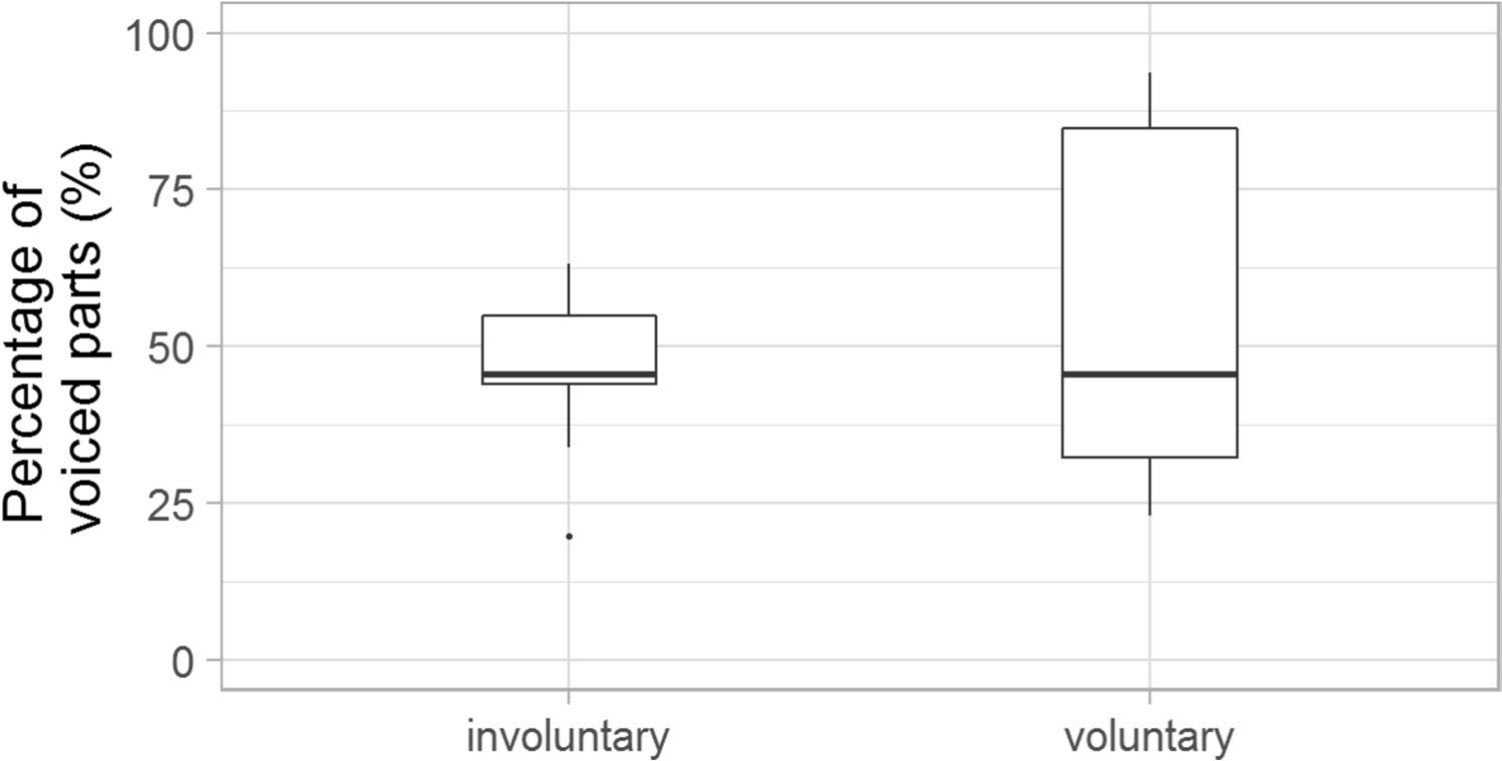
The ratio of the voiced parts in the total duration

The ratio of the voiced parts of the total duration was also analysed in the case of voluntary and involuntary laughs, categorised uniformly by the majority of the listeners. The voluntary laughter was characterised by a larger standard deviation of this value (mean: 63%, SD: 42%); however, no significant difference was found between laughter types according to the ratio of voiced parts (mean: 49%, SD: 9%). (Fig. 6).

**Fig. 7:**
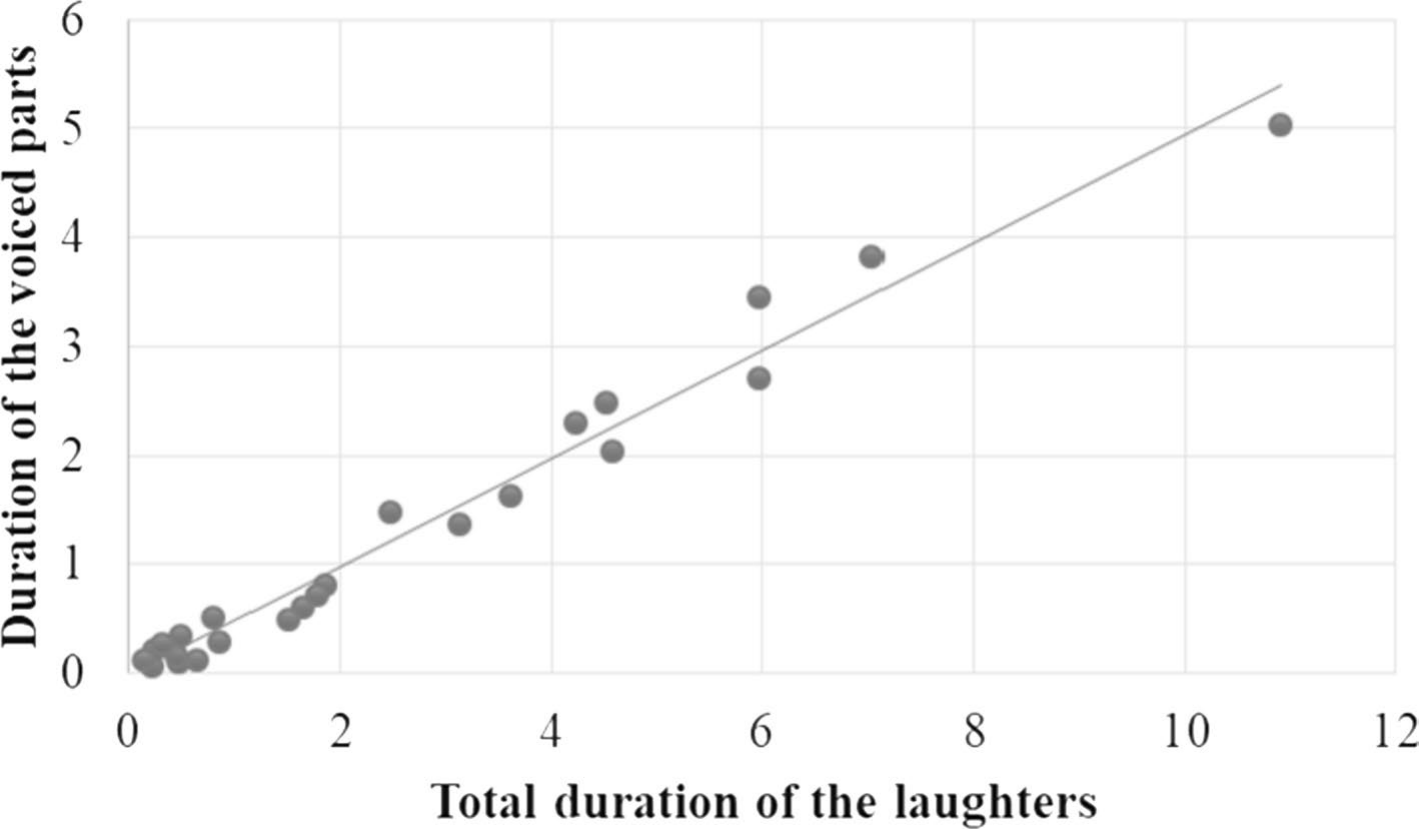
Correlation between the total duration (s) of laughter and the duration (s) of the voiced part

There was also a clear correlation (*r* = 0.62,* p* = 0.04) between the duration of the voiced parts and the duration of the laughter: the longer the duration of the laughter was, the greater the proportion of voicedness was (Fig. 7).

**Fig. 8:**
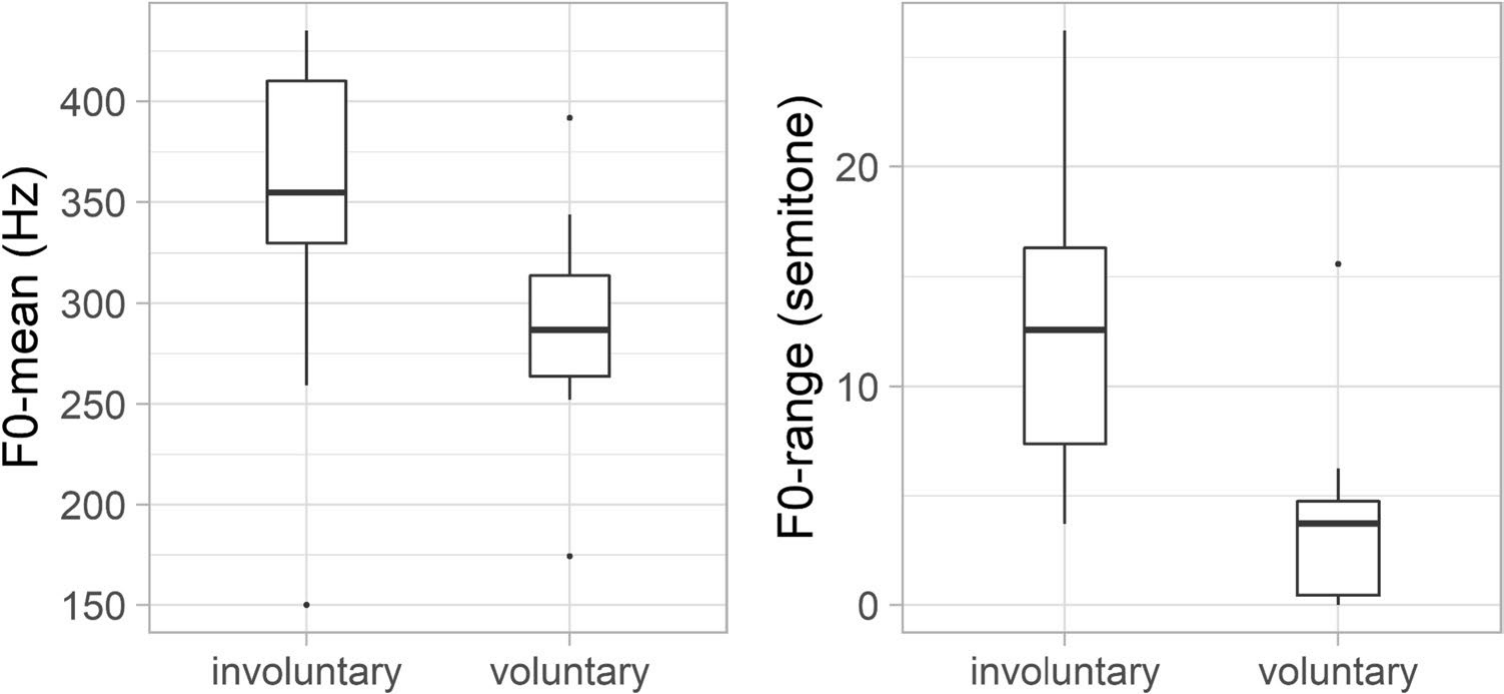
The f0 mean (left) and the f0-range (right) of the laughter

Fundamental frequency was higher (mean: 352 Hz) in the case of laughter that were determined to be involuntary by the majority of the participants, than the voluntary ones (mean: 288 Hz). The difference between the two laughter categories was significant: *Z* = – 2.331;* p* = 0.020 (Fig. 8).

This difference was found not only in the mean values of f0, but also in the f0 range (Fig. 6). The mean of the f0 range was 4.1 in the case of voluntary laughter (SD: 4.9), while 12.6 of involuntary ones (SD: 6.0). Involuntary laughter was characterised by significantly greater f0 variability (*Z* = − 3.087; *p* = 0.002) than voluntary laughter. There was a positive correlation between the timing characteristics and the f0 values in both groups: the listeners considered the laughter with longer duration (*r* = 0.52, *p* = 0.019) and higher f0 to be more involuntary (*r* = 0.47, *p* = 0.031).

**Fig. 9:**
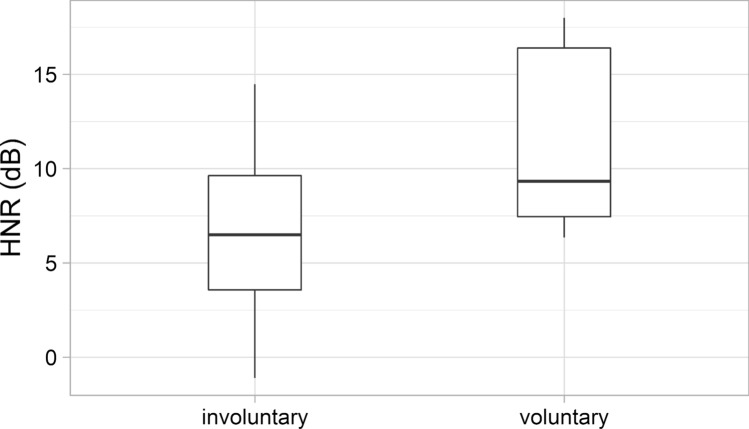
The HNR values of the involuntary and voluntary laughter

The HNR values were analysed and compared to the case of voluntary/involuntary laughs (categorised uniformly by at least 66.6% of all participants). The HNR values were somewhat higher in laughs that were considered to be voluntary than in the case of involuntary ones; the difference was not significant (Fig. 9). The mean of HNR was 11.03 dB (SD: 4.74 dB) at voluntary laughs, while 6.24 dB (SD: 4.22 dB) at involuntary ones.

**Fig. 10:**
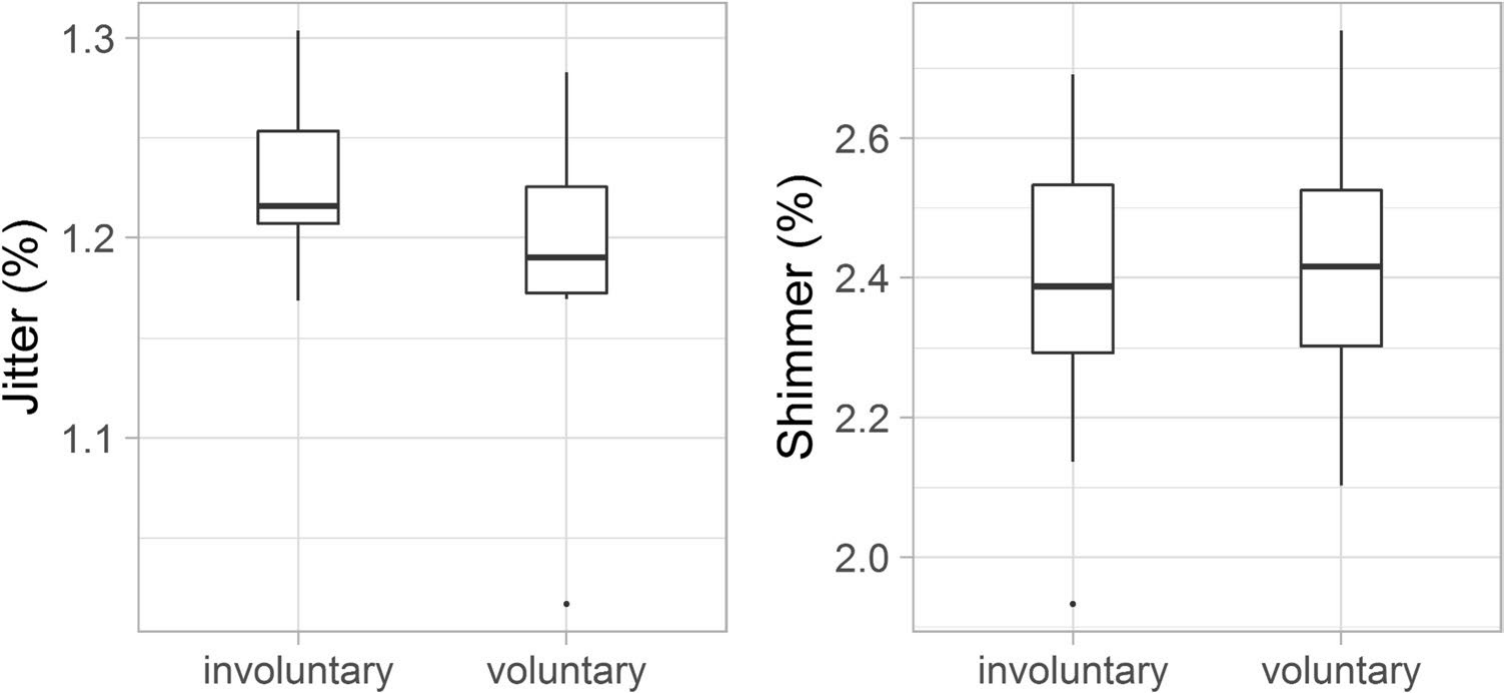
The jitter (left) and the shimmer (right) of the laughter

Lower jitter values were found; more regular vocal vibrations were considered in laughs that were categorised as voluntary (mean 1.18% SD: 0.07%) than in the case of involuntary ones (mean 1.22% SD: 0.04%). However, statistical analysis did not show any significant difference between the two groups Moreover, in this respect, shimmer showed no difference between laughter deemed to be involuntary (mean 2.37 SD: 0.2%) and voluntary (mean 2.43% SD: 0.2%, see Fig. 10).

**Fig. 11:**
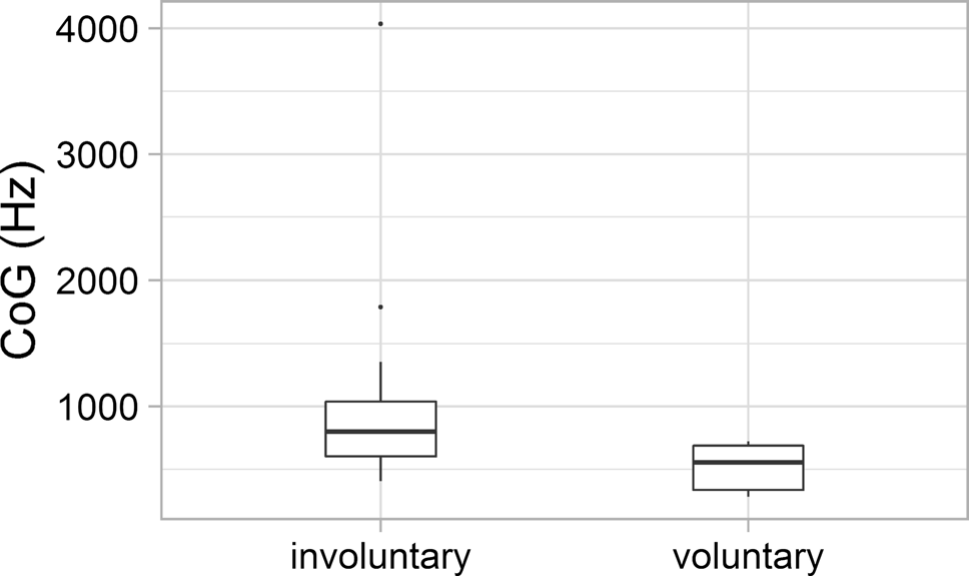
The CoG values of the involuntary and voluntary laughter

The mean centre of gravity (CoG) was 1048 (SD: 540) Hz in involuntary laughter and 517 (SD: 167) in voluntary laughter. The difference between the two laughter types was significant (*Z* = − 2.385;* p* = 0.017 (Fig. 11).

**Fig. 12:**
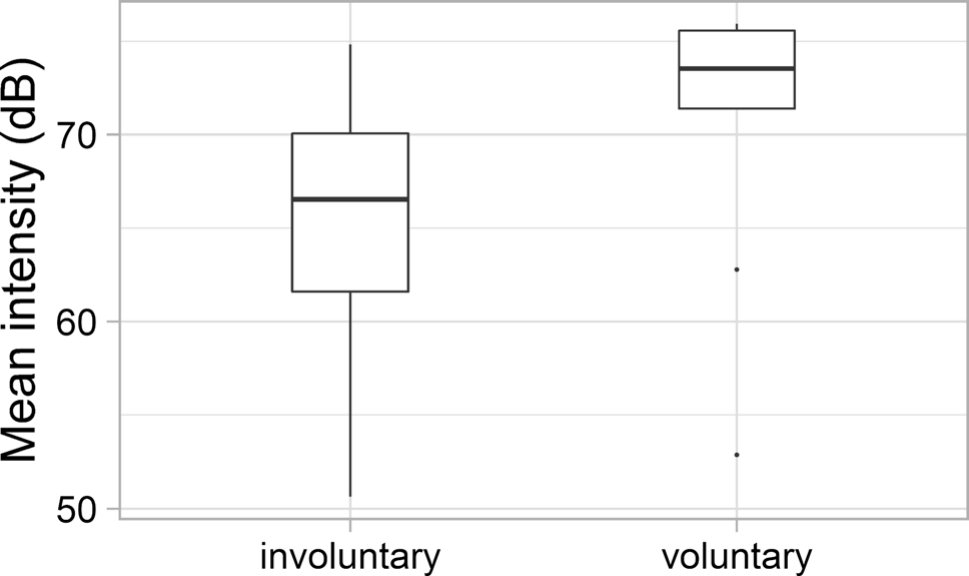
The mean intensity (dB) values of the involuntary and voluntary laughter

Finally, we measured the intensity of voluntary and involuntary laughter (Fig. 12). Involuntary laughter showed lower-intensity values, on average (mean: 64.8, SD: 5.9 dB), than voluntary laughter (mean: 70.4, SD: 5.6 dB). The two laughter types differed significantly in this feature as well (*Z* = − 2.206; *p* = 0.027).

Summarising the acoustic features of laughs considered to be voluntary/involuntary by the majority of the listeners; involuntary ones occurred with significantly longer duration, higher F0 mean, higher F0 variability than voluntary ones (Table 1).

**Table 1:** Acoustic parameters of laughter regarding voluntariness (categorised by large part of listeners)

Acoustic parameters of laughter regarding voluntariness		
	Involuntary	Voluntary
Mean duration (ms)	924 ± 455	520 ± 338
Mean f0 (Hz)	352 ± 77	288 ± 61
Mean f0 range (semitones)	12.6 ± 6.0	4.1 ± 4.9
Mean number of syllables (bursts)	3.4 ± 1.6	3.4 ± 1.2
Ratio of the voiced parts of the total duration (%)	47 ± 11	55 ± 28
Mean HNR (dB)	6.24 ± 4.22	11.03 ± 4.74
Mean jitter (%)	1.23 ± 0.04	1.19 ± 0.07
Mean shimmer (%)	2.38 ± 0.20	2.44 ± 0.22
Mean CoG (Hz)	1048 ± 540	517 ± 167
Mean intensity (dB)	64.8 ± 5.9	70.4 ± 5.6

Acoustic analysis was carried out on laughter which were not categorised uniformly by the listeners with regard to voluntariness (compound category) as well. The mean duration was 795 ms (SD: 423 ms) of laughter that was longer than the voluntary, and it was shorter than the involuntary ones. However, the difference was not significant.

The f0 parameters of laughter belonging to this compound category were also analysed. Data showed that the f0 mean of these laughter was very similar to laughter considered to be voluntary (289 Hz, SD: 76 Hz) by the majority of the participants. However, it was significantly lower than in the case of involuntary ones (*Z* = − 2.342; *p* = 0.019).

Similar to the duration values, the f0 range of laughter of the compound category occurred between the values of voluntary and involuntary categories (mean: 8.7 semitones, SD: 6.1 semitones). The difference was significant between the values of voluntary laughs and the laughs in the compound category (*Z* = − 2.225; *p* = 0.024).

Some parameters of voice quality were also analysed in the case of laughter, whose judgement was not so unanimous by the participants. The HNR values in the compound category occurred between the values of voluntary and involuntary laughter (mean: 8.8 SD: 4.8), similar to the duration and f0 range values. However, the jitter values were the highest in the compound category (mean: 1.24% SD: 0.04%) as well as the shimmer values (mean: 2.46% SD: 0. 3%).

### Automatic classification

**Fig. 13:**
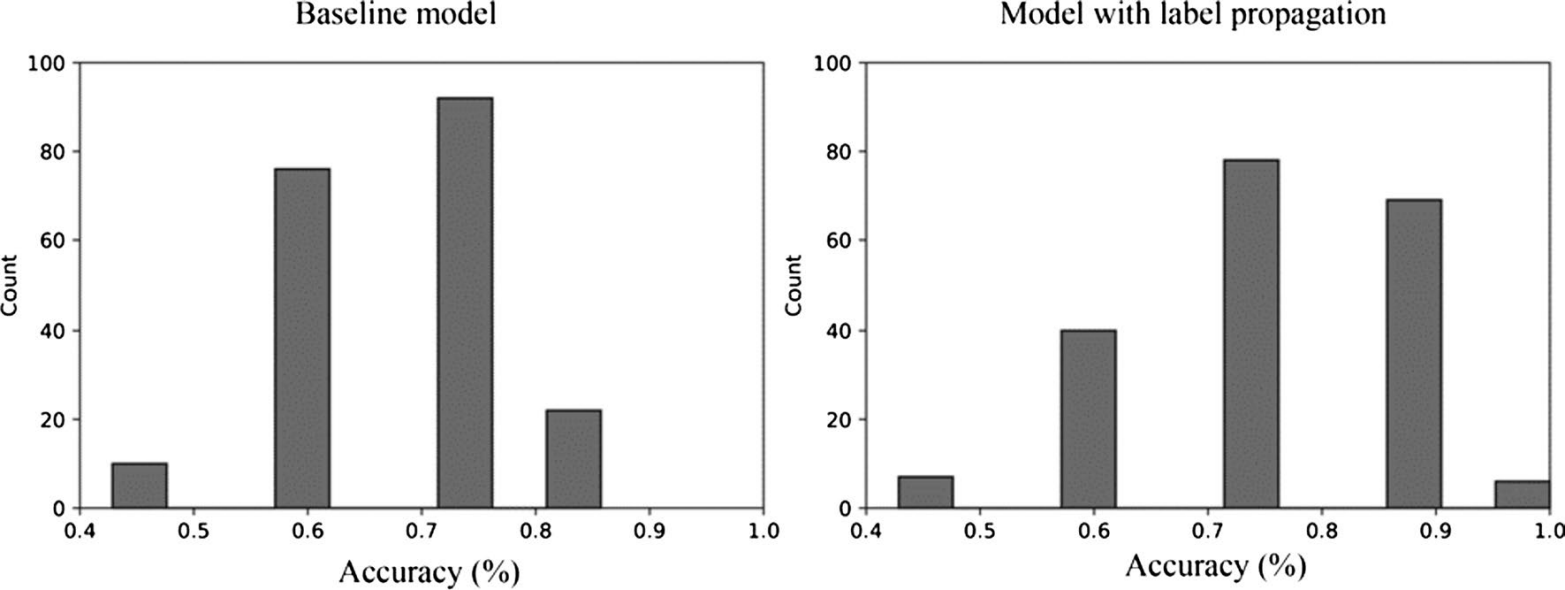
Distribution of accuracy of logistic regression models

Two logistic regression models were built: one of them was trained only on the labelled laughter samples (baseline); the other was trained on labelled and unlabelled data as well (with label propagation). The results demonstrated that both models performed properly. An accuracy of greater than 50% was obtained in 93.5% of cases (187 of 200 runs) for the baseline model and in 98% of cases (196 of 200 runs) for the model with label propagation. In both cases, the performance was significantly greater than 50% (baseline model: *t* = 18.466; *df* = 199; *p* < 0.001; label-prop model: *t* = 27.257; *df* = 199; *p* < 0.001). The models did not show remarkable bias towards classifying laughs as voluntary or involuntary. It is worth noting that both models had a wide range of accuracy. The performance of the logistic regression classification (Fig. 13) showed an improvement over the baseline when label propagation was used. The mean accuracy (Table 2) of the baseline model relying only on human-labelled laughter was 66.14%, whilst the model incorporating a larger set of unlabelled laughter was found to be 73.36% accurate, on average. This indicates that a 10.9% improvement could be achieved in this task using a semi-supervised technique. There was a significant difference between the performance of the two models (*t* = 6.159; *df* = 398; *p* < 0.001). The classification accuracy at the quartiles of the distribution showed a displacement to higher values (the accuracy scores are skewed towards the right side) for the model trained on a larger dataset compared to the baseline. It can also be seen in the distributions that the worst performance yielded the same values of accuracy in both tasks: 42.86%. The best performance of the baseline model yielded an accuracy of 85.71%, while the maximum value of 100% could be achieved in the model with label propagation.

**Table 2:** Performance of the logistic regression classification

	Baseline model (%)	Model with label propagation (%)
Mean	66.14	73.36
Std.	10.65	12.70
Min.	42.86	42.86
25% (Q1)	57.14	71.43
50% (Q2)	71.43	71.43
75% (Q3)	71.43	85.71
Max.	85.71	100.00

**Fig. 14:**
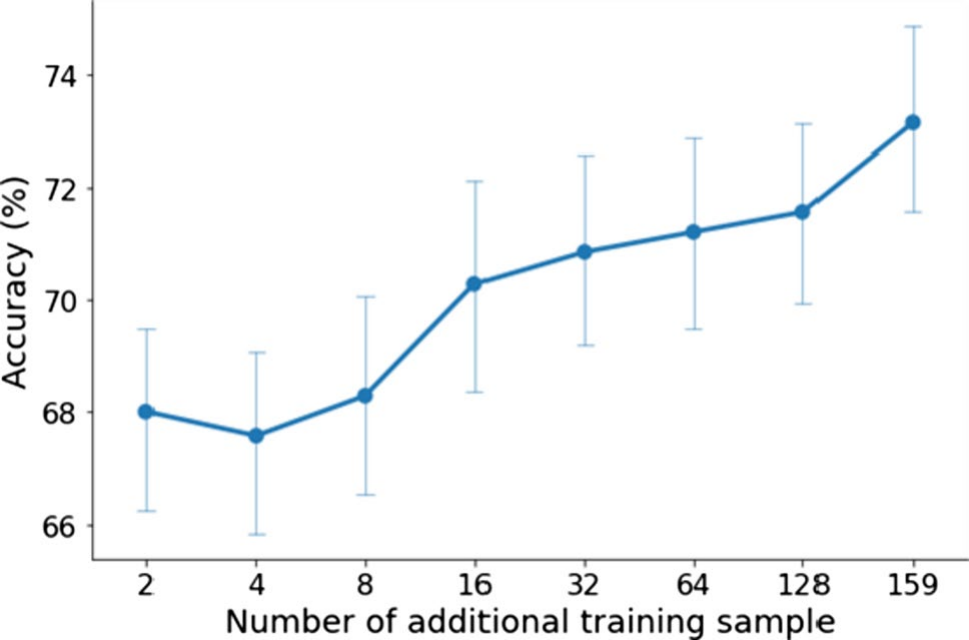
Accuracy (mean and SD) as a function of the number of additional training sample

Finally, we checked whether the label propagation works well on a piece of the additional dataset. The number of additional samples was increased along the powers of 2. That means, in the first case, we only added 2 unlabelled laughter to the train set. Then, we continued with the sample addition up to 128. The final train set contained all the 159 unlabelled laughter. The mean accuracy results of each logistic regression model showed a stronger improvement in performance when 16 samples were added and then in the transition from 128 to 159 (Fig. 14).

## Conclusions

Although non-verbal speech features, including laughter, were not the focal point of research in recent decades, it has now become one of the cornerstones, for example, in robotics and artificial intelligence (e.g. Due 2019; Hietalahti 2021). Several studies have shown that there is a difference between the two groups of laughs depending on why they are produced, and we can distinguish between natural/mirthful/real and polite/social/fake laughter (e.g. Shochi et al. 2017; Bryant et al. 2018; Sabonytė 2018) or during funny videos (Kamiloğlu et al. 2021). However, notable differences can be found in how the experiments defined these laughter and what kind of methodologies they use to record them. Recent research was mainly based on voluntary/involuntary laughter collected from experimental situations, focusing on these two types of laughs to be clearly distinguished (e.g. Bryant and Aktipis 2014; Lavan et al. 2016; Kamiloğlu et al. 2021). However, laughter mainly occurs in social situations in everyday communication, e.g. in conversations. In addition, there is little knowledge about how the two groups of laughter occur and are distinguished based on one of the most natural ways of the human, face-to-face communication, in spontaneous discussions. The question arose how voluntary and involuntary laughter can be differentiated based on laughter from face-to-face three-party conversations, and if so, what kind of acoustical differences can be found between the two groups. Therefore, the aim of the present study was to investigate whether listeners are able to differentiate laughter in natural conversations regarding the voluntariness of laughter. Laughter must have been categorised without their context. Results showed that listeners were able to make a discrimination between laughter regarding voluntariness, even there were no bad or good answers in this experiment (because all the samples were selected from spontaneous conversations, not from an experimental situation).

Great differences were found between the laugh samples with regard to their uniform categorisation of being involuntary or not. Half of the laughter samples were categorised uniformly by 50–66% of all participants. Thirty-eight percentage of the samples were categorised more uniformly (by 67–80% of the participants); only 10% of the laughs were deemed uniformly regarding voluntariness by at least 80% of the listeners.

Furthermore, the reaction time of the categorisation showed great variability across laugh samples and also across participants. The mean normalised reaction time was about 4 ms in the case of laughs categorised uniformly by at least 66.6% of the listeners, irrespective of whether they were deemed to be voluntary or involuntary.

Significant acoustic differences were found between laughter deemed to be voluntary and involuntary by the majority of listeners. Involuntary laughter (based on the categorisation of min. 66.6% of all listeners) was found to be longer than voluntary laughter, similar to previous studies (e.g. Bachorowski et al. 2001; Vettin and Todt 2004; Lavan et al. 2016; Shochi et al. 2017). Furthermore, significant differences were found in this study with regard to some f0 parameters. The mean fundamental frequency was higher and the f0 showed greater variability as well in the case of laughter that were considered to be involuntary by the majority of the participants, than the voluntary ones. These differences are in line with previous studies as well (e.g. Vettin and Todt 2004; Bryant and Aktipis 2014; Lavan et al. 2016; Shochi et al. 2017). Furthermore, the HNR was higher in the case of laughter deemed to be voluntary than involuntary ones; however, the difference was not significant, similar to the results of Lavan et al. (2016). The mean intensity of involuntary laughter was significantly lower than that of voluntary laughter—as also found by Lavan et al. (2016) acoustic analysis. More energy in the higher frequencies (higher CoG) was linked to involuntary laughter—similarly to the study of Lavan et al. (2016).

The acoustic analysis of this study merely provides an explicit characterisation of what the listeners consider as prototypes of voluntary/involuntary laughter. Therefore, data provide information of the listeners’ representation of laughter types in everyday communication. Laughter occurred with longer duration, higher f0 mean and f0 range in a conversation was perceived as involuntary phenomena rather than voluntary. In contrast, laughter with lower f0 and shorter duration in a conversation was rather categorised to be voluntary by the listeners.

Furthermore, clearer, more regular, less noisy laughter was considered to be more voluntary, noisier laughter was considered to be more involuntary—similar to Lavan et al.’s (2016) results; however, the difference was not significant in their study.

People are able to differentiate these two types of laughter not only in experimental situations, but in conversations as well. This result confirms the existence of strong cues about the characteristics of prototypes of voluntary/involuntary laughter. In other words, the different types of laughter are likely to have prototypical representations in communication based on prior experience and communicative prior knowledge, to which we can compare the often very diverse laughter that occurs in all situations.

The results of the automatic classification of laughter types support the validity of the judgments of the listeners in the perception test. There was 66.6% agreement among human listeners on the selected laughter samples. The average classification accuracy of the logistic regression model could be improved from 66 to 73% by adding unlabelled laughter to the train set and using the label propagation. Our results are in line with results of previous studies. Similarly to the acoustic analysis and automatic classification of two main types of laughter (polite and mirthful) in the study of Tanaka and Campbell (2014), we confirmed significant differences between voluntary and involuntary laughter in several acoustic parameters (e.g. duration and frequency features, in line with the study cited), and we also found that automatic classification of voluntary and involuntary laughter is possible at rates greater than chance (obtaining better than 70% of accuracy).

The limitation of the study should be noted: small amounts of labelled and unlabelled data from spontaneous speech were used for the study. This feature may result in an unstable model because of the small dataset. For further research, speaker-independent or speaker-dependent variables should be analysed as well as other techniques, such as transfer learning or few-shot learning, should be used. All these results suggest that the two types of laughter (*voluntary* and *involuntary*) can be distinguished based on their specific acoustic characteristics by human listeners and acoustic models as well, even without their context. However, beside the ‘prototypical’ involuntary and voluntary laughs, in the half of the cases, uncertain judgments were found regarding voluntariness in this study. Fifty-two percentage of all laughter samples were categorised uniformly less than 67% of all participants. In other words, in the half of the laugh samples, it was relatively hard to decide whether it was involuntary or not: given laughter sounded to be involuntary for the half participants, while rather voluntary for the other half of the participants. Some acoustic parameters of laughter in this compound category occurred with values between the voluntary and involuntary laughter’s. They occurred with longer duration, greater f0 variability and lower HNR value than laughs considered to be voluntary by the majority of the participants. In addition, the laughs that resulted in uncertain judgments compared to the involuntary category that occurred with shorter duration, less f0 variability and higher HNR value as well. The jitter and shimmer were found to be the highest in the case of laughs, which were not categorised unanimously by the participants. These results suggest that besides the acoustic parameters of laughter, listeners' own perception and previous experience might also affect the judgments, additionally some learned peculiarities.

The results of the research are limited, among other things, by the methodological procedure chosen. The number of laughter samples used in the perception test was relatively small, and only a subset of them in unbalanced categories was used in the production test. The reasons for this are, on the one hand, that previous studies have examined a similar number of samples and, on the other hand (closely related to this), that the attention and patience of the listeners is limited during the perception test, so that if too many sound samples are used, they either stop completing the test or pay less attention to answering, which calls into question the reliability of the results. To avoid this, we opted instead to process a sample with a smaller number of items. In addition, the inherent aim of the research was to make claims about the laughter from spontaneous conversations and to ensure that the categorisation of laughter was not based on a preconceived classification. Due to the combined effect of these factors, i.e. that the laughs were often non-prototypical non-verbal utterances, the distribution of the characteristics studied showed notable differences (e.g. the distribution of voluntary and involuntary laughs). However, the results of the research showed that similar results to previous research using other methodologies were found in the present study, although there was a significantly greater overlap between the two groups in both the perception of laughter and the analysis of acoustic characteristics.

In addition, the sample used in the study was based on randomly selected laughs. The aim was to get an idea of the perception of real, spontaneous, multi-person conversational laughter, as opposed to recording pre-categorised or other artificially generated (rather than prototypical) laughter in specific speech situations.
